# Eicosapentaenoic acid-rich oil supplementation activates PPAR-γ and delays skin wound healing in type 1 diabetic mice

**DOI:** 10.3389/fimmu.2023.1141731

**Published:** 2023-06-09

**Authors:** Beatriz Burger, Roberta Nicolli Sagiorato, Jéssica Rondoni Silva, Thamiris Candreva, Mariana R. Pacheco, Daniel White, Bianca G. Castelucci, Laís P. Pral, Helena L. Fisk, Izadora L. A. Rabelo, Jefferson Elias-Oliveira, Wislei Riuper Osório, Silvio Roberto Consonni, Alessandro dos Santos Farias, Marco Aurélio Ramirez Vinolo, Claudiana Lameu, Daniela Carlos, Barbara A. Fielding, Martin Brunel Whyte, Fernando O. Martinez, Philip C. Calder, Hosana Gomes Rodrigues

**Affiliations:** ^1^ Laboratory of Nutrients and Tissue Repair, School of Applied Sciences, University of Campinas, Limeira, Brazil; ^2^ Department of General Surgery, The Royal Surrey National Health Service (NHS) Foundation Trust Hospital, Guildford, United Kingdom; ^3^ Department of Biochemistry and Tissue Biology, Institute of Biology, University of Campinas, Campinas, Brazil; ^4^ Laboratory of Immunoinflammation, Department of Genetics, Evolution, Microbiology and Immunology, Institute of Biology, University of Campinas, Campinas, Brazil; ^5^ School of Human Development & Health, Faculty of Medicine, University of Southampton, Southampton, United Kingdom; ^6^ Department of Biochemistry, Institute of Chemistry, University of São Paulo, São Paulo, Brazil; ^7^ Departments of Biochemistry and Immunology, Ribeirão Preto Medical School, University of São Paulo, Ribeirão Preto, Brazil; ^8^ Laboratory of Manufacturing Advanced Materials, School of Applied Sciences, University of Campinas, Limeira, Brazil; ^9^ Autoimmune Research Lab, Department of Genetics, Evolution, Microbiology and Immunology, Institute of Biology, University of Campinas, Campinas, Brazil; ^10^ Department of Nutritional Sciences, Faculty of Health and Medical Sciences, University of Surrey, Guildford, United Kingdom; ^11^ Department of Medicine, King’s College Hospital National Health Service (NHS) Foundation Trust, London, United Kingdom; ^12^ Department of Clinical & Experimental Medicine, School of Biosciences and Medicine, University of Surrey, Guildford, United Kingdom; ^13^ Department of Biochemical Sciences, School of Biosciences and Medicine, University of Surrey, Guildford, United Kingdom; ^14^ National Institute for Health and Care Research (NIHR) Southampton Biomedical Research Centre, University Hospital Southampton National Health Service (NHS) Foundation Trust and University of Southampton, Southampton, United Kingdom

**Keywords:** tissue repair, diabetes, chronic wounds, nutrition, inflammation, fatty acids

## Abstract

Delayed wound healing is a devastating complication of diabetes and supplementation with fish oil, a source of anti-inflammatory omega-3 (ω-3) fatty acids including eicosapentaenoic acid (EPA), seems an appealing treatment strategy. However, some studies have shown that ω-3 fatty acids may have a deleterious effect on skin repair and the effects of oral administration of EPA on wound healing in diabetes are unclear. We used streptozotocin-induced diabetes as a mouse model to investigate the effects of oral administration of an EPA-rich oil on wound closure and quality of new tissue formed. Gas chromatography analysis of serum and skin showed that EPA-rich oil increased the incorporation of ω-3 and decreased ω-6 fatty acids, resulting in reduction of the ω-6/ω-3 ratio. On the tenth day after wounding, EPA increased production of IL-10 by neutrophils in the wound, reduced collagen deposition, and ultimately delayed wound closure and impaired quality of the healed tissue. This effect was PPAR-γ-dependent. EPA and IL-10 reduced collagen production by fibroblasts *in vitro*. *In vivo*, topical PPAR-γ-blockade reversed the deleterious effects of EPA on wound closure and on collagen organization in diabetic mice. We also observed a reduction in IL-10 production by neutrophils in diabetic mice treated topically with the PPAR-γ blocker. These results show that oral supplementation with EPA-rich oil impairs skin wound healing in diabetes, acting on inflammatory and non-inflammatory cells.

## Graphical Abstract

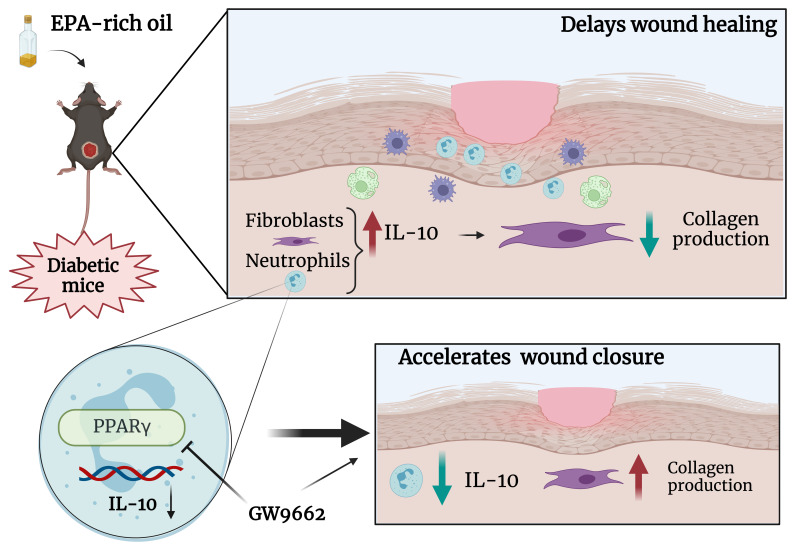

## Introduction

Hard-to-heal wounds are common among patients living with diabetes mellitus. Such wounds show delayed, interrupted or stalled healing (1) and are the main cause of lower limb amputation in patients with diabetes (1). The main mechanisms involved in this include exacerbated inflammation, delay in angiogenesis, dysfunction in collagen organization and consequent impairment in extracellular matrix (ECM) formation, abnormalities in fibroblast and keratinocyte migration and proliferation, and loss of antioxidant capacity (2). Despite the clinical burden of this problem, there is no effective treatment for hard-to-heal wounds for in patients with diabetes.

Fatty acids alter skin structure and immunological status since they are present in all skin layers modulating cell function and production of eicosanoids, reactive oxygen species and cytokines. Consequently, the inflammatory response and wound healing process are affected by fatty acids (3–10). Omega-3 (ω-3) fatty acids can be obtained from several sources such as fish, some vegetable oils, nuts (especially walnuts), flax seeds, flaxseed oil and leafy vegetables. However, fish are the predominant dietary source of eicosapentaenoic acid (EPA, C20:5ω-3) and docosahexaenoic acid (DHA, C22:6ω-3), which are also available in supplements such as fish oil capsules (11).

Despite the widely considered health benefits of omega-3 fatty acids, some studies have demonstrated certain adverse consequences of these compounds in wound healing (8, 12–15). It has been reported that omega-3 fatty acids, mainly the combination of DHA and EPA, substantially delay the wound healing process and interrupt collagen organization in healthy mice by different pathways (8, 15). The actions of omega-3 fatty acids include multiple mechanisms involving alterations in membrane organization, formation of lipid mediators, as well as activation of intracellular receptors that drive alterations in gene expression (16).

Among these receptors, the peroxisome proliferator-activated receptors (PPARs) are responsive to omega-3 fatty acids (17, 18). These transcription factors belong to a nuclear receptor superfamily, which includes three isotypes PPAR-α, PPAR-β/δ and PPAR-γ (19), involved in regulation of gene expression in a variety of cells and tissues, including inflammatory cells and skin (20). For example, there is extensive literature demonstrating that EPA acts partly through PPAR-γ to alter anti-inflammatory responses (21–23).

In this study, we have tested the effects of oral administration of EPA-rich oil on skin wound healing in diabetic mice.

## Materials and methods

### Mice

The Ethical Committee for Animal Research of the University of Campinas (UNICAMP, Brazil) approved the protocols used in the present study (CEUA – 4975-1/2018; 5786-1/2021). All experiments with mice were carried out at the School of Applied Sciences (UNICAMP) and followed the National Institute of Health guidelines for the use of experimental mice. C57Bl6 male mice were purchased from the UNICAMP Animal Breeding Center.

C57Bl6 mice (8-weeks-old) were divided into three groups: control (C) mice supplemented with water; streptozotocin (STZ)-induced diabetes (D) mice supplemented with water; and STZ-induced diabetes mice supplemented with EPA-rich oil (ED) for 4 weeks (**Supplementary Figure 1A**).

The protocol of multiple low doses of STZ is a widely used model to induce insulin-dependent type 1DM. The drug has high affinity to pancreatic β-cells, where it induces DNA fragmentation and cell death, resulting in reduction in insulin production (24).

The standard chow (Nuvital; Curitiba, Brazil) contained 64.6% unsaturated and 35.4% saturated fatty acids. Among the unsaturated fatty acids, only 1.8% consisted of EPA, as previously reported (4). Considering the habitual food ingestion of the mice (4 g/day) (**Supplementary Figure 1B**), the dose supplemented in the present study represented an increase of approximately 75% in the amount of omega-3 fatty acids ingested.

### Diabetes induction and oral administration of EPA-rich oil

Diabetes was induced over 5 consecutive days by intraperitoneal (i.p.) injection of 45 mg/kg of streptozotocin (S0130, Sigma-Aldrich^®^) diluted in citrate buffer (pH 4.2) (25, 26). Mice in the control group (C) received citrate buffer injections. After 10 days, initial glycemia was measured using an AccuChek active glucometer (Roche, Mannheim, Germany) and mice were considered diabetic when glycemia was greater than 240 mg/dL.

EPA-rich oil was supplied by the Naturalis^®^ Company (São Paulo, Brazil). A dose of 2 g/kg body weight (50 μL) was administered by gavage daily during four weeks, as described previously (8). Control mice received 2 g/kg body weight of water by gavage, considering that the amount of the oil administrated represented only 1.98 kcal/day (27).

### Omega-3 supplement and serum fatty acid composition

The protocol used for analysis of the composition of fatty acids in EPA-rich oil capsules and serum was adapted from the methodology described previously (28). Mouse blood was collected by cardiac puncture 3 days after wound induction. Blood was centrifuged at 4,481g for 15 minutes at 4°C to yield serum. Methanolic NaOH (1 mL) and 25 uL internal standard (1,2-dipentadecanoyl-sn-glycero-3-phosphocholine, Sigma^®^, 850350P, diluted to 1mg/mL in isooctane) were added to 150 uL serum or oil capsules. This mixture was heated for 15 minutes. After cooling, 2 mL esterification reagent (boron trifluoride-methanol solution, BF3, Sigma^®^, B1252) was added and the samples were heated for 5 minutes. Then, 1 mL isooctane and 5 mL saturated NaCl solution was added. After separation of the phases, the upper phase (250 uL) was collected for analysis [see (15) for detailed methodology]. Identification of fatty acid peaks was made by comparison with the 37 fatty acid methyl ester calibration standards.

### Skin fatty acid composition

Skin samples were homogenized with chloroform:methanol (2:1 vol/vol) using a polytron PT 1200 Kinematica (Lucerne, Switzerland) for total lipid extraction, as described elsewhere (29). Phosphatidylcholine (PC), phosphatidylethanolamine (PE), non-esterified fatty acid (NEFA), cholesteryl ester (CE) and triacylglycerol (TAG) fractions were isolated from the skin lipid extracts by solid-phase extraction on aminopropylsilica cartridges (Sep Pak C18 Cartridges, Waters^®^, Milford, MA, USA) [see (8, 29) for detailed methodology]. The total omega-6 fatty acid content was calculated as the sum of the concentrations of linoleic (18:2ω-6), γ-linolenic (18:3ω-6), eicosadienic (20:2ω-6), di-homo-γ-linolenic (20:3 ω-6) and arachidonic (20:4ω-6) acids. Total omega-3 fatty acid content was calculated as the sum of α-linolenic (18:3ω-3), eicosatetraenoic (20:4ω-3), eicosapentaenoic (20:5ω-3), docosapentaenoic (22:5ω-3) and docosahexaenoic (22:6ω-3) acids.

### Wound induction and measurement

Prior to wound induction, mice were anesthetized with xylazine and ketamine solution (2:1 v:v). Hair was removed using a razor and a mold of 1 cm^2^ skin (dermis and epidermis) was surgically-removed from the dorsal region. Mice were sacrificed 1, 3, 7 and 10 days after wound induction by inhalation with isoflurane (12%). For evaluation of closure, the wounds were photographed daily. After digitalization, *ImageJ* software^®^ (National Institute of Health, Bethesda, MD) was used for wound area measurement. The reduction of wound area, expressed as percentage (%) of the original area, was used as an indication of wound closure.

### Histological analysis

Skin samples (100 mg) were fixed in formaldehyde 4% diluted in 0.1 M phosphate-buffered saline (PBS; pH 7.4) for 24 hours at 4°C. Next, the samples were processed and 5 μm slices mounted on slides and stained with hematoxylin/eosin for structural analysis. Sirius Red staining combined with polarized light detection was used to evaluate collagen fibre organization (30), following the protocol described by Candreva et al. (15).

### Wound tensile strength measurements

A universal electromechanical machine with computerized brand control (Equilam^®^, model WDW 100E (Brazil, Diadema-SP)) was used for mechanical tests. For uniaxial traction analysis, wound samples (100 mg) were collected from the mice 10 days after wound induction and sectioned in the longitudinal direction. Edges and wounds of 20 ± 1 mm (length) x 5 ± 0.5 mm (width) x 0.5 ± 0.02 mm (thickness) were considered.

A schematic representation of the prepared samples is shown in **Supplementary Figure 2**. The displacement beam speed was about 1 mm/minute. A strain rate of approximately 2.5 x 10^-4^s^-1^ at room temperature 27 ± 2 °C was applied. These measurements were carried out at least in duplicate. The mechanical parameters were evaluated considering the ultimate tensile strength (UTS), in Mega Pascal (MPa), and specific elongation (%).

### Quantitative reverse transcription PCR

Total mRNA was extracted from wound tissue (60 mg) using the RNeasy Mini Kit (Qiagen, Venlo, The Netherlands). Trizol^®^ (Invitrogen Corporation, CA, USA) was used for RNA extraction from cultured neutrophils and the High-Capacity cDNA Reverse Transcription kit (Applied Biosystems, Foster City, CA) was used for cDNA synthesis. Reactions were performed using QuantiNova SYBR-Green PCR Kit (Quiagen^®^) or iTaq Universal SYBR Green Supermix (1725121, Bio-Rad^®^) in a StepOnePlus™ RT-qPCR System. Ubiquitin C (UBC) and beta-2-microglobulin (B2M) were used as endogenous controls. The delta-delta cycle threshold (ΔΔCt) method was used to calculate fold changes (31). **Supplementary Table 1** shows the primer sequences used for the RT-qPCR experiments.

### Determination of cytokine concentrations in wound tissue

Wound tissues (100 mg) were removed at 0 hours and 1, 3, 7 and 10 days after wound induction and processed as described (8). Duo Set ELISA kits (R&D System^®^, Minneapolis, MN, USA) were used for determination of cytokines [IL-1β, tumor necrosis factor alpha (TNF-α), chemokine C-X-C motif ligand 1 (CXCL-1), IL-6 and IL-10], collagenase matrix metalloproteinase-9 (MMP-9), tissue inhibitor of metalloproteinase (TIMP-1) and vascular-endothelial growth factor (VEGF) concentrations. Protein concentrations were determined by the method of Bradford for normalization purposes (32).

### *In situ* zymography

Seven days after wound induction, wounds were collected and frozen in isopentane under liquid nitrogen. The reactions were performed using 5 μm cryosections as described (33). The sections were mounted with VECTASHIELD Mounting Medium (Invitrogen, Waltham, Massachusetts, EUA) and visualized with a fluorescence microscope (Zeiss-Axio Scope.A1) using a 20 x objective.

### Phenotypic characterization of leukocytes by flow cytometry

The expression of cell markers in wound tissue was evaluated by flow cytometry. Tissues (100 mg) were collected 7 and 10 days after wound induction and samples were processed as described previously (8). Non-reactive labeled IgG antibody was used as a negative control. Samples were analyzed on a BD FACSymphony™ A5 Cell Analyzer and measurements performed using FlowJo Software 10 (BD Bioscience). One hundred thousand events were acquired per sample. Percentage of leukocytes was determined from CD45 positive (CD45+) cells and that of non-leukocytes was determined from CD45 negative (CD45-) cells.

### *Ex vivo* flow cytometry

Single-cell suspensions of wound cells were plated in flat bottom ultra-low attachment surface polystyrene Costar^®^ 24 well plates, with Dulbecco’s Modified Eagle Medium (DMEM) medium containing 10% fetal bovine serum (FBS) for 30 minutes. GolgiStop™ (1:1.500 dilution - BD Biosciences; Cat. 51-2092KZ) was added to stop Golgi-mediated protein transport. Next, phorbol 12-myristate 13-acetate (PMA; 500 ng/mL), ionomycin (500 ng/mL) and lipopolysaccharide LPS (1 μg/mL) were added to stimulate cytokine production. After 4 hours, cells were washed and processed for flow cytometry (34). Cells were stained with extracellular antibodies (**Supplementary Table 3**), fixed with 2% formaldehyde and then washed/permeabilized with triton X-100 (0.1%) and intracellular staining performed. The samples were analyzed by flow cytometry using FlowJo Software 10 as described above.

### Peritoneal neutrophil culture

Thioglycolate 4% was injected (i.p.) in diabetic mice. Four hours later, 5 mL Roswell Park Memorial Institute (RPMI) medium containing 5% FBS was used to obtain the intraperitoneal lavage. Cells were centrifuged (2,987 g for 10 minutes at 4°C), washed in 5 mL erythrocyte lysis solution and centrifuged again. Cells (1 x 10^6^/well) were plated in 24-well plates and incubated with LPS (1 μg/mL), EPA (5, 10, 25, 50 μM) or the PPAR-γ antagonist GW9662 (10 μM; M6191, Sigma-Aldrich^®^) for 24 hours. The supernatant and cells were used in the analysis. Cells were used for RT-qPCR to determine the gene expression of *Ppar-γ*, interleukin-10 receptor (*IL-10R)*, G-protein coupled receptor 120 *(Gpr-120)*, histamine receptor 1 *(H1R)* and *H2R.*

### Primary dermal fibroblast culture

The protocol was adapted from Almeida et al. (35). Approximately 160 mg skin from 3 different mice, was digested for 1 hour in DMEM containing 0.1% type IV collagenase solution (Sigma Aldrich, Saint Louis, MO, USA) and 20% FBS at 37°C, under agitation (20 rpm). Next, samples were centrifuged (2,987 g, 10 minutes, 4°C) and resuspended in DMEM with 15% FBS for plating. During 7-15 days, cells were maintained at 37°C in a 5% CO_2_ incubator. The medium was replaced every two days. We used 1-7 passages for all experiments. For the experiments, cells were maintained in high glucose medium (30 mM D-glucose) treated with EPA (50 µM) or IL-10 (10 ng/mL).

### Calcein AM staining and spectroscopic analysis

Neutrophils (1 x 10^6^/well) or fibroblasts (6 x 10^3^/well) were seeded and treated with EPA. After 24 hours of EPA exposure, the medium was removed and the cells incubated with 50 μL 1 μM Calcein AM and Hoetchst (1µg/mL) for 30 minutes at 37 °C under 5% CO_2_. Cell viability was measured by absorbance at 495/516 nm using a microplate reader (36).

### Determination of cytokine concentration in cell supernatants

The supernatant of cells was collected for quantification of cytokine and other relevant mediators as described above.

### *In vivo* pharmacological inhibition of PPAR-γ

After diabetes induction, EPA-rich oil supplementation and wound induction, mice were divided into two groups: (ED+PBS) diabetic mice supplemented with EPA-rich oil and topically-treated with 100 μL PBS (vehicle); and (ED+GW9662) diabetic mice supplemented with EPA-rich oil and topically-treated with 2 μM GW9662 in PBS over 10 days. The concentration of GW9662 was determined based on a description in a patent application (37). Wound closure was evaluated as described earlier and the tissues collected were used for *ex vivo* flow cytometry and for histological analyses.

### Statistical analysis

Results are presented as mean ± standard error of the mean and their normality was evaluated using the Shapiro-Wilk tests. As p value of Shapiro-Wilk were greater than 0.05, the data were considered normal.

Comparisons between groups were performed using one-way or two-way ANOVA and Bonferroni post-hoc tests as indicated in figure and table legends. Statistical analyses were performed using Prisma 8.0^®^ (GraphPad Software, Inc., San Diego, USA). Differences were considered significant for p <0.05. To analyze correlations between wound area (cm^2^) and wound IL-10 concentration, curve estimation models were used since these variables were not related linearly. The relationships were tested for logarithmic, quadratic, cubic, logistic, exponential, and S-shape regression models. The significant model (p < 0.05) with higher explanatory power (R²) was selected.

## Results

### EPA delayed wound closure and collagen production by fibroblasts

**Figure 1:**
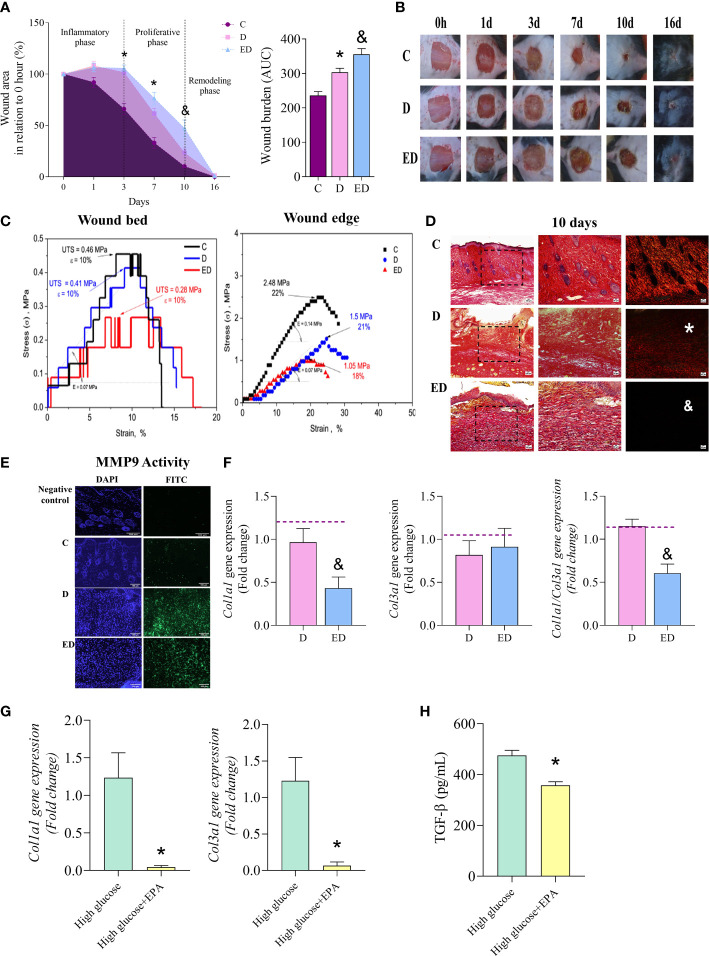
EPA delayed wound closure and collagen production by fibroblasts. **(A)** Percentage wound area in relation to initial area and wound burden (area under the curve-AUC) in control **(C)**, diabetic **(D)** and diabetic mice supplemented with EPA-rich oil (ED) for 4 weeks. Values are expressed as mean ± standard error of the mean (SEM). p <0.05 was considered statistically significant as indicated by two or one-way ANOVA and Bonferroni post-test. (*) C vs. D; (&) D vs. ED. Animals/group/day: 4-8C, 4-7D, 5-9ED. **(B)** Representative photographs of wounds after 0, 1, 3, 7, 10 and 16 days. **(C)** Typical tension curves and specific elongation measurements performed in wound bed and edge collected 10 days after induction (n: 2 C, 2 D, 2 ED, in 2 independent experiments). **(D)** Representative photomicrographic of Sirius Red staining 10 days after wound induction (animals/group: 5C, 5D and 5ED). Images were taken at magnifications with 10x (100 µm scale) and 20x (50 µm scale) lenses. (*) indicates scarce collagen fibres in D group in comparison to the C group and (& indicates scarce collagen fibres in ED group in comparison to the D group. **(E)** *In situ* zymography of MMP-9 activity. DAPI^+^ (blue) stains the nuclei and MMP-9 activity is stained with FITC^+^ (green). Animals/group: 2C, 6D, 5ED. **(F)** Gene expression of collagen 1a1 *(Col1a1)*, 3a1 *(Col3a1)* and ratio *Col1a1/Col3a1* in wound scar collected at 7 and 10 days after wounding (animals/group: 5-6D, 4-6ED). The median of *Ubc* and *β2m* expression was used as a housekeeping control. **(G)** *Collagen 1a1* and *3a1* gene expression in primary dermal fibroblasts treated with only high glucose medium (30 mM glucose) or high glucose medium and EPA (50 µM) for 7 days. **(H)** TGF-β concentration in supernatants of dermal fibroblasts treated with high glucose medium only or high glucose medium and EPA (50 µM) for 7 days (n: 6 High; 6 High+EPA, in 2 independent experiments). Values are expressed as mean ± SEM. p <0.05 was considered statistically significant as indicated by unpaired t test (&) D vs. ED. (*) High vs. High+EPA.

Diabetic mice had delayed wound closure at 3 and 7 days after wound induction compared to control, non-diabetic mice (**Figures 1A, B**). Supplementation of diabetic mice with EPA-rich oil delayed wound healing by day 10 in comparison to diabetic mice (**Figures 1A, B**), suggesting a deleterious effect of EPA on this model. Using area-under-the-curve analysis to determine wound burden, we found that wound closure was prolonged in the EPA-treated diabetic mice group (**Figure 1A**).

To evaluate effects on skin functionality, typical engineering tensile versus strain curves corresponding with the examined wound tissues are shown in **Figure 1C**. This showed that the lowest ultimate tensile strength (UTS) was that of the wound tissue collected from the EPA-treated diabetic mice (*i.e.* a 0.28 MPa was attained). When the control and diabetic groups are considered, the UTS values showed that the failure occurred at 0.46 and 0.41 MPa, respectively (**Figure 1C**). There were no verified differences (within 10%) in the attained elasticity values among the examined samples (**Figure 1C**). When the tensile strengths of the wound edges were analysed, both diabetic groups had reduced values in comparison to the control group. Again, the elasticity was not significantly affected, as shown in **Figure 1C**.

Diabetic mice displayed thin and parallel collagen fibres, contrasting with the thick and reticular fibre organization observed in control mice (**Figure 1D**). Supplementation with EPA-rich oil further aggravated this aspect, as determined by staining with Sirius Red which showed fewer collagen fibres in the EPA-treated diabetic mice (**Figure 1D**).

*In situ* zymography showed that the diabetic group had higher MMP-9 activity than the control mice (**Figure 1E**). Although there was no statistical difference between EPA-treated diabetic and the diabetic groups, MMP-9 activity was still intense in the former (**Figure 1E**). We also observed that EPA reduced collagen 1a1 (*Col1a1*) gene expression in comparison with the diabetic group (**Figure 1F**). This difference resulted in a reduction of *Col1a1/Col3a1* ratio in the diabetic mice treated EPA (**Figure 1F**). As collagen is produced by fibroblasts, we performed *in vitro* experiments with primary dermal fibroblast cultures and observed that fibroblasts maintained in high glucose medium and treated with EPA (50 µM) had diminished *Col1a1* and *Col3a1* gene expression (**Figure 1G**). Moreover, EPA treatment reduced the concentration of TGF-β in the supernatant of fibroblasts (**Figure 1H**). The toxicity of EPA was analyzed by the Calcein-AM assay and there were no significant differences in cell viability in cells treated with EPA (**Supplementary Figure 3**).

### The effect of EPA on wound healing was not attributed to nutritional modifications

The diabetic group presented higher glucose concentrations, as well as higher water and food intake than the control group, as expected (**Supplementary Figures 1A–C**). The effect of EPA on wound healing was not attributed to glycemia modifications, since no differences in blood glucose between the diabetic and EPA-treated diabetic groups were observed during the wound healing process (**Supplementary Figure 1C**). At the same time, nutritional parameters were not different between these two groups (**Supplementary Figures 1A, B**).

Serum and the skin fatty acid compositions were analyzed to investigate if diabetes and the EPA-rich oil supplementation could modify these. In serum, the diabetic group had a higher linoleic acid (18:2 ω-6) concentration than the control group and EPA and DHA were elevated in serum in the EPA-treated diabetic group in comparison to diabetic mice (**Supplementary Figure 4A**). These modifications resulted in reduction of the serum ω-6/ω-3 ratio in diabetic mice treated with EPA in relation to the diabetic mice (**Supplementary Figure 4B**).

In skin, diabetic mice had lower incorporation of linoleic acid and DHA, and a higher ω-6/ω-3 ratio compared to control mice in PC fraction (**Supplementary Figure 5A**). There was an increase in linoleic acid, α-linolenic acid (C18:3, ω-3) and DHA in PC and PE fractions in the EPA-treated diabetic group compared to diabetic mice (**Supplementary Figures 5A, B**). As expected, we observed a decrease in the ω-6/ω-3 ratio in the PE fraction in EPA-treated diabetic mice compared to the diabetic group (**Supplementary Figure 5B**). The modifications in fatty acid composition of NEFA, CE and TAG did not result in alteration of the ω-6/ω-3 ratio in these fractions (**Supplementary Figures 5C–E**).

We also analyzed the composition of EPA-rich oil and found that 71.35% of the oil was EPA, followed by 19.65% DHA, 0.22% linolenic acid, and 8.78% others. Omega-3 fatty acids constituted 91.39%, omega-6 fatty acids 6.02%, omega-9 fatty acids 1.73% and saturated fatty acids 0.83% of the oil (**Supplementary Table 2**).

### EPA-rich oil reduced the inflammatory response induced by diabetes in the proliferative phase

**Figure 2:**
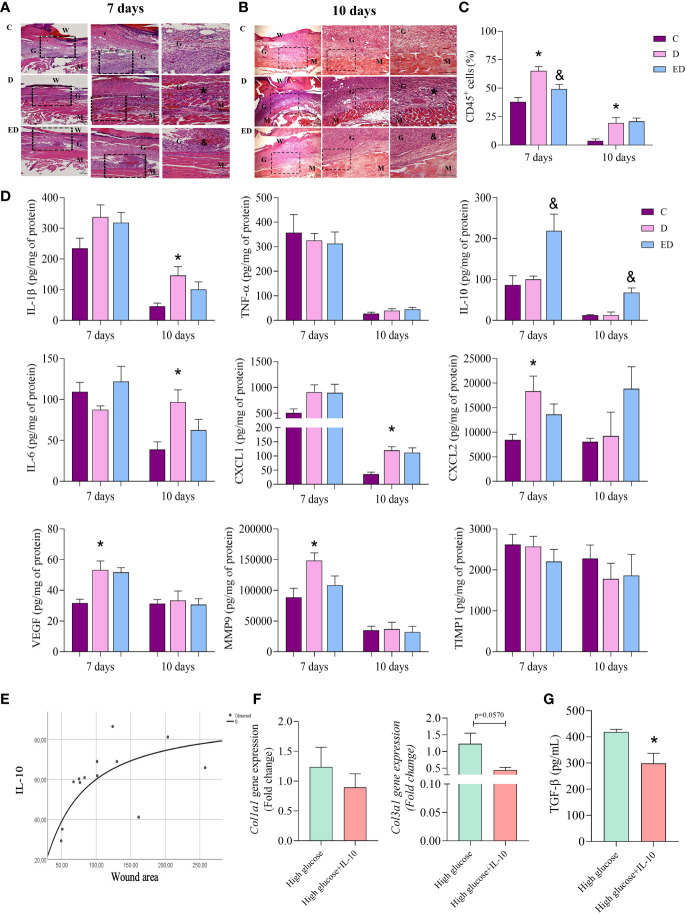
EPA-rich oil reduces the inflammatory response induced by diabetes in the proliferative phase. **(A, B)** Samples were collected after 7 and 10 days of wound induction from control **(C)**, diabetic **(D)** and diabetic mice supplemented with EPA-rich oil (ED). To histological analysis, the samples were stained with hematoxylin and eosin (animals/group: 5C, 5-6D, 4-5ED). Photomicrographs were taken at magnifications with 10x and 20x lenses. Legend: W=Wound; G = Granulation tissue; M = Muscle layer; (*) indicates presence of inflammatory infiltrates on muscular layer of D group in comparison to C group. (&) indicates reduction of inflammatory infiltrates in the ED group in comparison to the D group. **(C)** Flow cytometry of wound tissue 7 and 10 days after wound induction. Numbers of leucocytes (CD45^+^) were measured. Animals/group: 4-5C, 5D, 4-5ED. **(D)** Tissue concentrations of IL-β, TNF-α, IL-10, IL-6, CXCL1, CXCL2, VEGF, MMP9 and TIMP1 measured at 7 and 10 days after wound induction. Animals/group/day: 4-6C, 4-8D, 4-9ED. Values are expressed as mean ± SEM. p <0.05 was considered statistically significant as indicated by one-way ANOVA and Bonferroni post-test. (*) C vs. D; (&) D vs. ED. **(E)** Correlation between wound area and wound IL-10 concentration shows the S-shape regression model. R² = 0.49; p=0.007. **(F)** *Collagen 1a1* and *3a1* gene expression in primary dermal fibroblasts treated with only high glucose medium alone or high glucose medium and IL-10 (10 ng/mL) for 7 days. **(G)** TGFβ concentration in supernatant of cells. (n: 6 High; 6 High+IL-10, in 2 independent experiments). Values are expressed as mean ± SEM. p <0.05 was considered statistically significant as indicated by unpaired t test (*) High vs. High+IL-10.

Diabetic mice presented an increase in cell infiltration and oedema in the wound during the proliferative phase (7 and 10 days after wound induction) in comparison to the control group (**Figures 2A, B**). Using flow cytometry, we demonstrated that these were inflammatory cells which expressed CD45 (**Figure 2C**). In addition, the EPA-treated group showed reduced infiltration of inflammatory cells at the wound site in relation to diabetic mice at both time-points evaluated (**Figures 2A, B**). By flow cytometry, we also confirmed this reduction day 7 after wound induction (**Figure 2C**).

At 7 days after wound induction, no differences were observed in concentrations of IL-1β, TNF-α, IL-10, IL-6 and CXCL1 between the diabetic and control mice. However, together with the increase in inflammatory cells, diabetic mice had higher concentrations of CXCL2, VEGF and MMP-9 at this time point. At 10 days, the diabetic mice showed an increase of IL-1β, IL-6 and CXCL1 in comparison to the control group (**Figure 2D**). Interestingly, the EPA-treatment of the diabetic mice led to increased IL-10 concentrations compared to the diabetic mice that had not been treated with EPA at both time points and no other differences were observed between these two groups (**Figure 2D**). In addition, no differences were observed in TIMP1 concentration (**Figure 2D**).

### EPA-rich oil increased the production of IL-10 by neutrophils in the proliferative phase of wound healing

Considering that EPA supplementation to diabetic mice increased IL-10 concentrations, the correlation between IL-10 and wound area was investigated. The best fit curve tested was the S-shape (R² = 0.49; p=0.007). This demonstrated an initial increment, followed by a stationary phase (**Figure 2E**). Based on this result, we investigated the effects of IL-10 (10 ng/mL) on primary fibroblast cultures. IL-10 did not modify the expression of *Col1a1* but we observed a trend (p=0.057) in reduction of *Col3a1* expression in fibroblasts treated with IL-10 (**Figure 2F**). In culture supernatants, we observed that IL-10 reduced TGF-β levels (**Figure 2G**).

**Figure 3:**
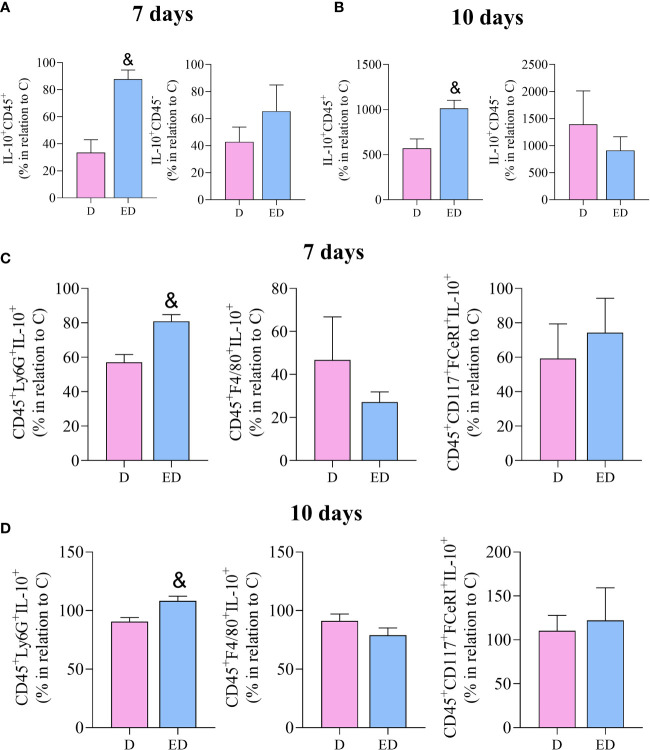
EPA-rich oil increases the production of IL-10 by neutrophils in the proliferative phase of wound healing. **(A, B)** Percentage of IL-10 positive leucocytes (IL-10^+^CD45^+^) and non-immune cells (IL-10^+^CD45^-^) after 7 and 10 days of wound induction in diabetic **(D)** and diabetic mice supplemented with EPA-rich oil (ED). **(C, D)** Neutrophils (IL-10^+^CD45^+^Ly6G^+^), macrophages (IL-10^+^CD45^+^F4/80^+^) and mast cells (IL-10^+^, CD45^+^CD117^+^FceRI^+^) quantified by flow cytometry after 7 and 10 days of wound induction. Animals/group: 4-5D, 4-5ED. Values are expressed as mean ± SEM. p <0.05 was considered statistically significant as indicated by unpaired t test. (&) D vs. ED.

To determine which cell type is the source of IL-10, *ex vivo* flow cytometry was performed. This showed that EPA treatment resulted in an increase in the percentage of leukocytes that produce IL-10 (IL-10^+^CD45^+^) in relation to diabetic mice, at 7 and 10 days after wound induction. On the other hand, there was no difference between diabetic and EPA-treated diabetic mice in non-immune cells that were positive for IL-10 (IL-10^+^CD45^-^) (**Figures 3A, B**). Among the leukocytes, neutrophils (CD45^+^Ly6-G^+^IL-10^+^) were the main source of IL-10 in the diabetic mice treated with EPA compared to the diabetic group at both time points. No differences were observed in IL-10-producing macrophages (CD45^+^F4/80^+^IL-10^+^) or mast cells (CD45^+^CD117^+^FCeRI^+^IL-10^+^) (**Figures 3C, D**).

### EPA increased IL-10 through PPAR-γ activation in neutrophils

**Figure 4:**
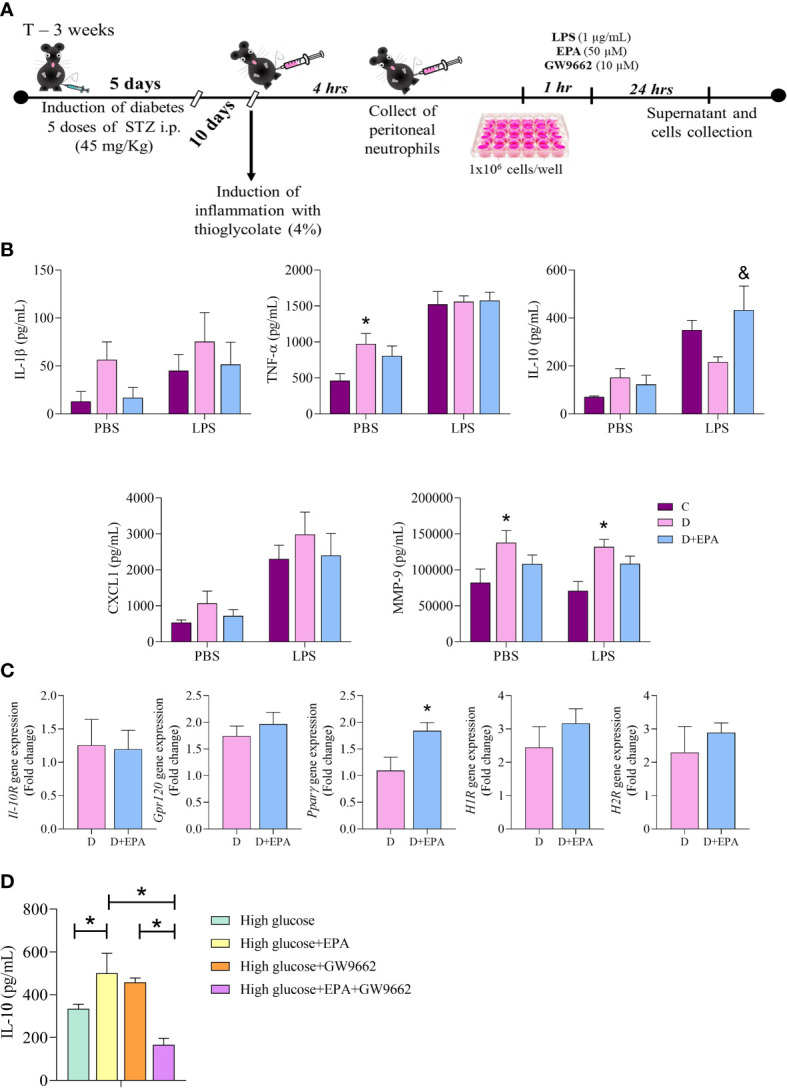
*In vitro* treatment with EPA induces IL-10 production through PPAR-γ activation in neutrophils. **(A)** Primary cell culture experimental design. After diabetes induction with 5 doses of streptozotocin (STZ), peritonitis was induced by injection of thioglycolate (4%). Neutrophils were collected after 4 hours and stimulated with LPS (1 µg/mL), EPA (50µM) or GW9662 (10µM). The supernatants and cells were collected after 24 hours. **(B)** IL-1β, TNF-α, IL-10, CXCL1 and MMP9 concentration in the supernatant of neutrophils harvested from control, **(C)**, diabetic mice **(D)** and neutrophils collected from diabetic mice and treated *in vitro* with EPA (D+EPA) in the absence (PBS) and presence of 1 µg/mL LPS. Animals/group: 5C, 4-5D, 4-5D+EPA. Values are expressed as mean ± standard error of the mean (SEM). p <0.05 was considered statistically significant as indicated by two-way ANOVA and Bonferroni post-test. (*) Difference in relation to C (&) Difference in relation to D C Il-10R, Gpr120, PPARγ, H1R and H2R gene expression in neutrophils. Values are expressed as mean ± SEM. p <0.05 was considered statistically significant as indicated by unpaired t test. (*) Difference in relation to D. **(D)** The IL-10 concentration in the supernatant of neutrophils treated with high glucose medium, EPA (50 µM), GW9662 (10 µM) or EPA+GW9662. Values are expressed as mean ± SEM. p <0.05 was considered statistically significant as indicated by two-way ANOVA and Bonferroni post-test. (*) Indicates significantly differences. Animals/group: 6 High, 5 High+EPA, 5 High+GW9662, 9 High+EPA+GW9662.

Elicited neutrophils isolated from the peritoneal cavity of diabetic mice were stimulated with LPS and treated with EPA (**Figure 4A**). A concentration-response assay showed that 5, 10 or 25 µM EPA did not alter IL-10 production, but 50 µM EPA increased IL-10 production by neutrophils (**Supplementary Figure 6A**).

Neutrophils isolated from the diabetic group had higher production of MMP-9 and TNF-α than those from the control group. These cytokines were not affected by *in vitro* EPA (50 µM) treatment (**Figure 4B**). The EPA treatment increased IL-10 concentrations in relation to the diabetic mouse group after LPS stimulation (**Figure 4B**). *In vitro* treatment with EPA increased *Ppar-γ* mRNA in neutrophils with no alterations observed in *IL-10R, Gpr-120, H1R* and *H2R* gene expression (**Figure 4C**). Next, we investigated the effects of the specific PPAR-γ inhibitor (GW9662) in cultured neutrophils and found a decrease in production of IL-10 by EPA-treated neutrophils (**Figure 4D**). The potential toxicity of EPA in neutrophils was analyzed by the Calcein-AM assay and this showed that there were no significant differences in cell viability in cells treated with different concentrations of EPA (**Supplementary Figure 6B**).

### Pharmacological inhibition of PPAR-γ reversed the effects of EPA on wound healing in diabetic mice

**Figure 5:**
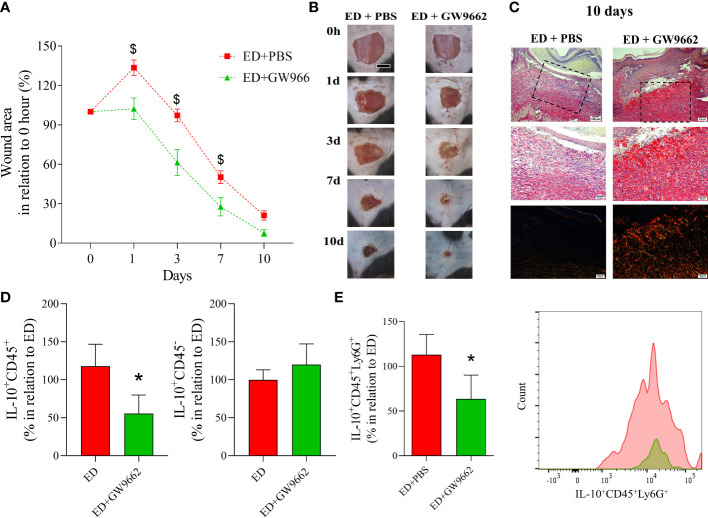
Topical application of PPAR-γ antagonist reverses the deleterious effects of EPA-rich oil supplementation on wound healing in diabetic mice. **(A)** Wound area percentages and representative photographs at 1, 3, 7 and 10 days from diabetic mice supplemented with EPA-rich oil and topically treated with PBS (ED+PBS) and diabetic mice supplemented with EPA-rich oil and topically treated with GW9662. Values are expressed as mean ± SEM. p <0.05 was considered statistically significant as indicated by two-way ANOVA and Bonferroni post-test. ($) Difference in relation to ED+PBS. Animals/group: 7 ED+PBS, 6 ED+GW9662. **(B)** Representative photographs of wounds at times 0, 1, 3, 7 and 10 after wounding. **(C)** Representative photomicrographs of Sirius Red staining 10 days after wound induction. Images were taken at 10x and 20x magnification. Animals/group: 4ED+PBS, 4ED+GW9662. **(D)** Percentage of IL-10 positive leucocytes (IL-10^+^CD45^+^) and non-immune cells (IL-10^+^CD45^-^) after 10 days of wound induction. **(E)** Neutrophils (IL-10^+^CD45^+^Ly6G^+^) quantified by flow cytometry after 10 days of wound induction with representative histograms. Values are expressed as mean ± SEM. p <0.05 was considered statistically significant as indicated by unpaired t test. (*) ED+PBS vs. ED+GW9662. (*) Difference in relation to ED+PBS. Animals/group: 7ED+PBS, 5-6ED+GW9662.

Finally, we investigated if *in vivo* pharmacological inhibition of PPAR-γ reversed the effects of EPA-rich oil administration on wound healing in diabetic mice. This showed that topical application of GW9662 reversed the deleterious effects of EPA on diabetic mice that had been supplemented with the EPA-rich oil. Specifically, these mice had a reduction in wound area 1, 3 and 7 days after wound induction in relation to EPA-treated mice that had not received the GW9662 topical treatment (**Figures 5A, B**). Using Sirius Red staining, we found that the latter group presented thin and parallel collagen fibers, contrasting with the thick and reticular fiber organization observed in the EPA-treated mice that had also received the GW9662 treatment (**Figure 5C**). Moreover, flow cytometry analysis showed that *in vivo* PPAR-γ-inhibition decreased IL-10 production by CD45^+^ cells and neutrophils in these mice (**Figures 5D, E**).

## Discussion

Our study demonstrates that EPA supplementation adversely impacts wound healing in diabetic mice, through reductions in collagen gene expression, impaired collagen organization and consequential reduction in resistance of the repaired tissue. Mechanistically, EPA supplementation enhanced IL-10 production by neutrophils through PPAR-γ activation. At the same time, EPA and IL-10 reduced the expression of collagens and TGF-β production by fibroblasts. Potentially the most important finding was that the impairment in collagen organization induced by EPA was rescued by PPAR-γ blockade *in vivo* (Graphical Abstract).

Our findings also confirmed that the STZ low-dose model was marked by impaired wound healing, *via* modification of inflammation, as well as the synthesis of collagen and its organization, which leads to a disruption in the mechanical functions of repaired skin. Excess inflammation is believed to impair wound healing (38) although effective healing does involve an inflammatory phase. Thus, the anti-inflammatory properties of omega-3 fatty acids, including EPA (39), may influence wound healing. However, EPA supplementation had deleterious effects in this model, further slowing the wound closure rate, reducing collagen deposition and decreasing the strength of the healed tissue (8). These adverse effects of EPA have not been described so far in the context of diabetes.

Previous studies described beneficial and deleterious effects of omega-3 fatty acids on skin wound healing both in rats and in humans (7–9, 40). These discrepancies can be explained by the different composition (pure fatty acid or blend of fatty acids) and doses/concentrations of the oils used. Moreover, the time and form of treatment (topical, oral supplementation, enriched-diet) can influence the findings. Furthermore, placebos such as corn oil or mineral oil are not good options because they have effects on the immune system that could result in effects in the control groups (27).

Skin collagen disorder is a common characteristic in mice with STZ-induced diabetes, which was confirmed in the present study, and is related with poor skin quality in diabetic patients (41, 42). This characteristic can be explained by the reduction in expression of collagen genes combined with the increase in MMP-9 activity, as observed in the diabetic mice in the current study. Collagens, secreted by fibroblasts, are the main components of the ECM and contribute to the tensile strength of skin (43). As already mentioned, MMPs degrade collagen and TIMPs block this degradation, so the reorganization of collagen is orchestrated by the balance of these enzyme systems (42, 44). Considering our results, it is clear that EPA impaired collagen organization and reduced wound strength in STZ mouse model.

Tissue inflammation is modulated by the cellular lipid composition (45). Therefore, the increase in serum EPA and DHA content and reduction in the ω-6/ω-3 ratio in serum and skin after EPA supplementation, demonstrate the potential of EPA to modulate the local inflammatory response. Inflammation is an essential step for appropriate wound healing (46, 47) although this must be strictly regulated (48, 49). Based on this, the cellular profile and inflammatory mediators present at the proliferative phases of wound healing were characterized. Diabetic mice had reduced TNF-α and IL-6 concentrations in the inflammatory phase of healing. Furthermore, during the proliferative phase, diabetic mice had higher concentrations of CXCL2 (7 days) and IL-1β, IL-6 and CXCL1 (10 days) than the control group, showing a dysregulation in inflammation control. Chemokines are essential to orchestrate the inflammatory phase of the wound healing (50–52). Although our model showed that the diabetic mice presented a late inflammatory response with increase of CXCL1 and CXCL2 in the proliferative phase, the EPA-treated diabetic group did not show this change. These results suggest that the inflammatory response was increased in the diabetic group compared to the control group in the proliferative phase of wound healing. Additionally, supplementation with EPA-rich oil attenuated the inflammatory response by reducing the infiltration of inflammatory cells and by increasing IL-10 concentrations.

We observed an increase of IL-10 concentrations in the diabetic mice treated with EPA compared to the diabetic mice treated with water. IL-10 is a mediator that regulates inflammation to protect the organism from tissue damage induced by immune responses (53, 54). Previous studies have demonstrated that IL-10 plays a deleterious role during skin wound healing (8, 53, 55), suggesting that this cytokine is a negative regulator of healing during tissue repair. In the current study, the observed increase in IL-10 concentrations in the inflammatory and proliferative phases in the EPA-treated diabetic mice was associated with the delayed wound closure rate, as demonstrated by the correlation between IL-10 concentrations and wound area.

In view of the relevance of IL-10 for tissue repair, we investigated the source of this cytokine during the proliferative phase. Interleukin 10 is produced by various immune and non-immune cells (54). The current *ex vivo* flow cytometry analyses demonstrated that neutrophils were the main IL-10-producing cells in EPA-treated diabetic mice. In addition, we found that EPA activated the transcription factor PPAR-γ leading to induction of IL-10 production. We confirmed this finding using the pharmacological PPAR-γ inhibitor GW9662 which resulted in lower IL-10 production in the EPA-treated diabetic model. These results are in agreement with previous studies that demonstrated that omega-3 fatty acids increased the protein expression of PPAR-γ and reduced inflammatory molecules such as TNF-α, prostaglandin E_2_ and 12-HETE (56). At the same time, PPAR-γ activity was also increased in PBMCs isolated from patients with type 2 DM who were orally supplemented with DHA-enriched oil (18). Taken together, these results reinforce the mechanism involving PPAR-γ activation and induction of anti-inflammatory effects by EPA.

Several groups have investigated the anti-scarring mechanisms of IL-10, and some signaling pathways seems to be involved such as PI3K/AKT/STAT-3 (57, 58). However, there is a lack of a more wound-like model to confirm the IL-10–mediated regulation of MMPs and ultimately whether collagen formation is affected.

Based on the effects of EPA-rich oil supplementation upon collagen organization and IL-10 production, we evaluated collagen gene expression in primary dermal fibroblasts treated with EPA and recombinant IL-10. These experiments demonstrated that both EPA and IL-10 were able to reduce the expression of collagen genes by fibroblasts. In both conditions, there was also an inhibition in TGF-β gene expression. TGF-β is a well-established pro-fibrotic agent because it induces cell proliferation, migration, and fibroblast activation (59, 60). From these findings, it follows that EPA could reduce collagen expression by suppression of the TGF-β signaling pathway. We suggest that future studies should investigate this possibility.

## Conclusion

In the present study, we demonstrate that EPA-rich oil supplementation delays wound healing in diabetic mice through increased production of IL-10 and impaired collagen organization in the repaired tissue. This latter effect results in a less resistant tissue after wound induction. We also demonstrated that EPA increased IL-10 production through PPAR-γ activation in neutrophils and *in vivo* blockade of the PPAR-γ pathway reversed the deleterious effects of EPA-rich oil supplementation in the diabetic mice. This effect suggests that PPAR-γ inhibition can be a potential therapeutic target for treatment of impaired wound healing in people living with diabetes and who take omega-3 supplementation. Overall, the present study sheds light on the role of nutrition in treating wound healing during diabetes (Graphical Abstract).

## Data availability statement

The raw data supporting the conclusions of this article will be made available by the authors, without undue reservation.

## Ethics statement

The Ethical Committee for Animal Research of the University of Campinas (UNICAMP, Brazil) approved the protocols used in the present study (CEUA – 4975-1/2018; 5786-1/2021).

## Author contributions

Conceptualization: BB, HR. Investigation: BB, DW, RS, JS, TC, MP, BC, LP, HF, IR, JE-O, WO, SRC. Formal analysis: BB, AF, HR. Funding acquisition: HR. Methodology: BB, DW, BAF, MW, FM, MV, HR. Project administration: HR. Writing-original draft: BB, MV, PC, HR. Writing-review & editing: all authors. All authors contributed to the article and approved the submitted version.
