# Supplementary material for: Eicosapentaenoic acid-rich oil supplementation activates PPAR-γ and delays skin wound healing in type 1 diabetic mice
Source: Front Immunol. 2023 Jun 9;14:1141731. doi: 10.3389/fimmu.2023.1141731 (PMC10289002; doi:10.3389/fimmu.2023.1141731)
Supplement: Supplementary file 1 [file DataSheet_1.docx]

**Supplementary material**

**Results**

**
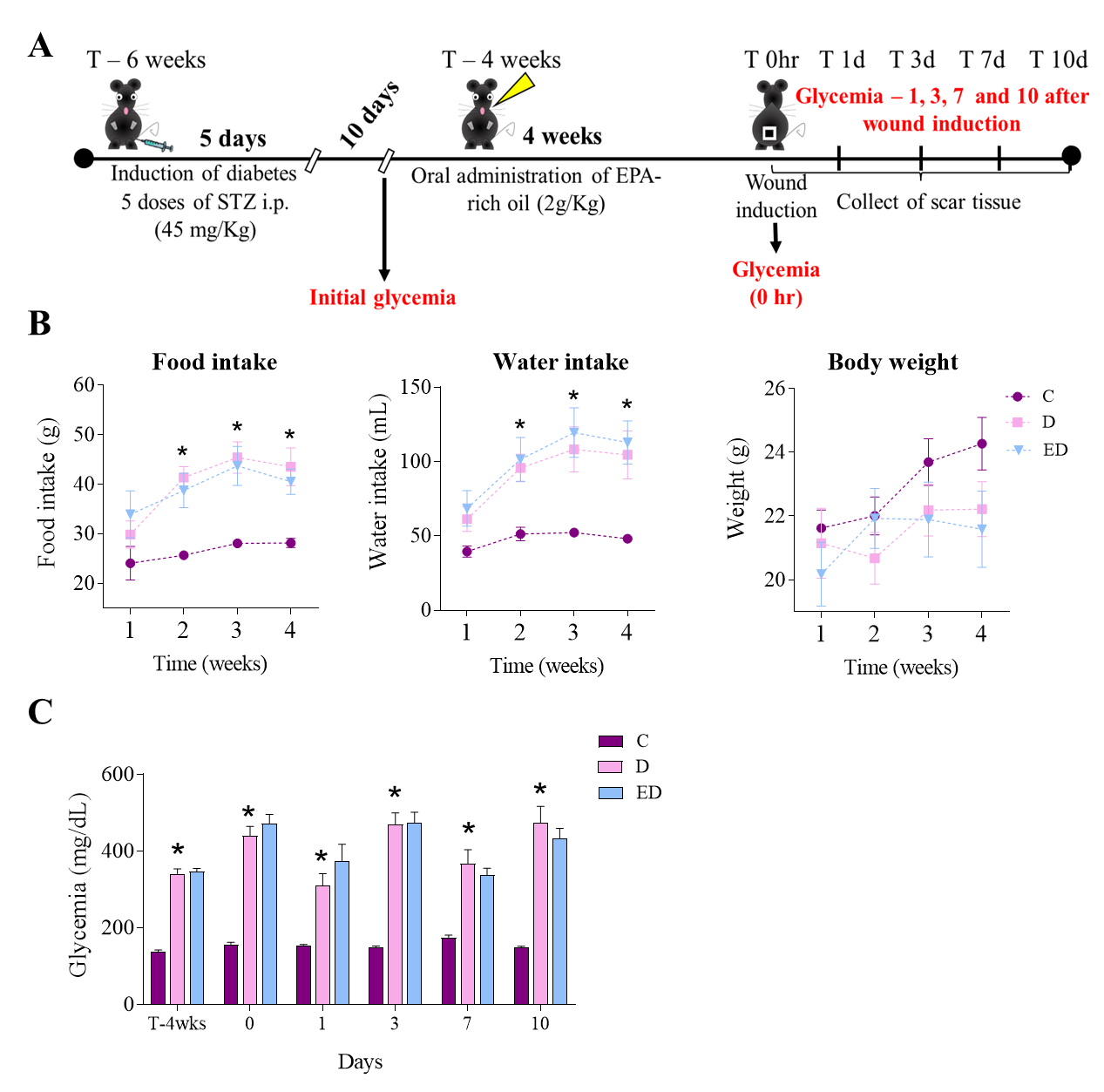
**

**Supplementary figure 1. Experimental design and nutritional parameters**. **(A)** Induction of diabetes for five consecutive days with 45 mg/kg of STZ. Ten days after diabetes was confirmed by a glycemic index above ≥240mg/dL. Upon diabetes confirmation, daily oral supplementation with EPA-rich oil was started. After 4 weeks, the wound was created and samples were collected at different times for analysis. **(B)** The food intake, water intake and body weight measured in control (C), diabetic (D) and diabetic mice supplemented with EPA-rich oil (ED). The analyses were performed once a week during 4 weeks. **(C)** Blood glucose was measured 10 days after induction of diabetes with STZ (T-4wks) and before wound induction (0) and after wound induction (1, 3, 7 and 10 days). Values ​​are expressed as mean ± standard error of the mean (SEM). p <0.05 was considered statistically significant as indicated by Two-Way ANOVA **(B)** or One-Way ANOVA **(C)** and Bonferroni post-test. (*) C *vs.* D. Animals/group: 4-8C, 4-7D, 5-9ED

**
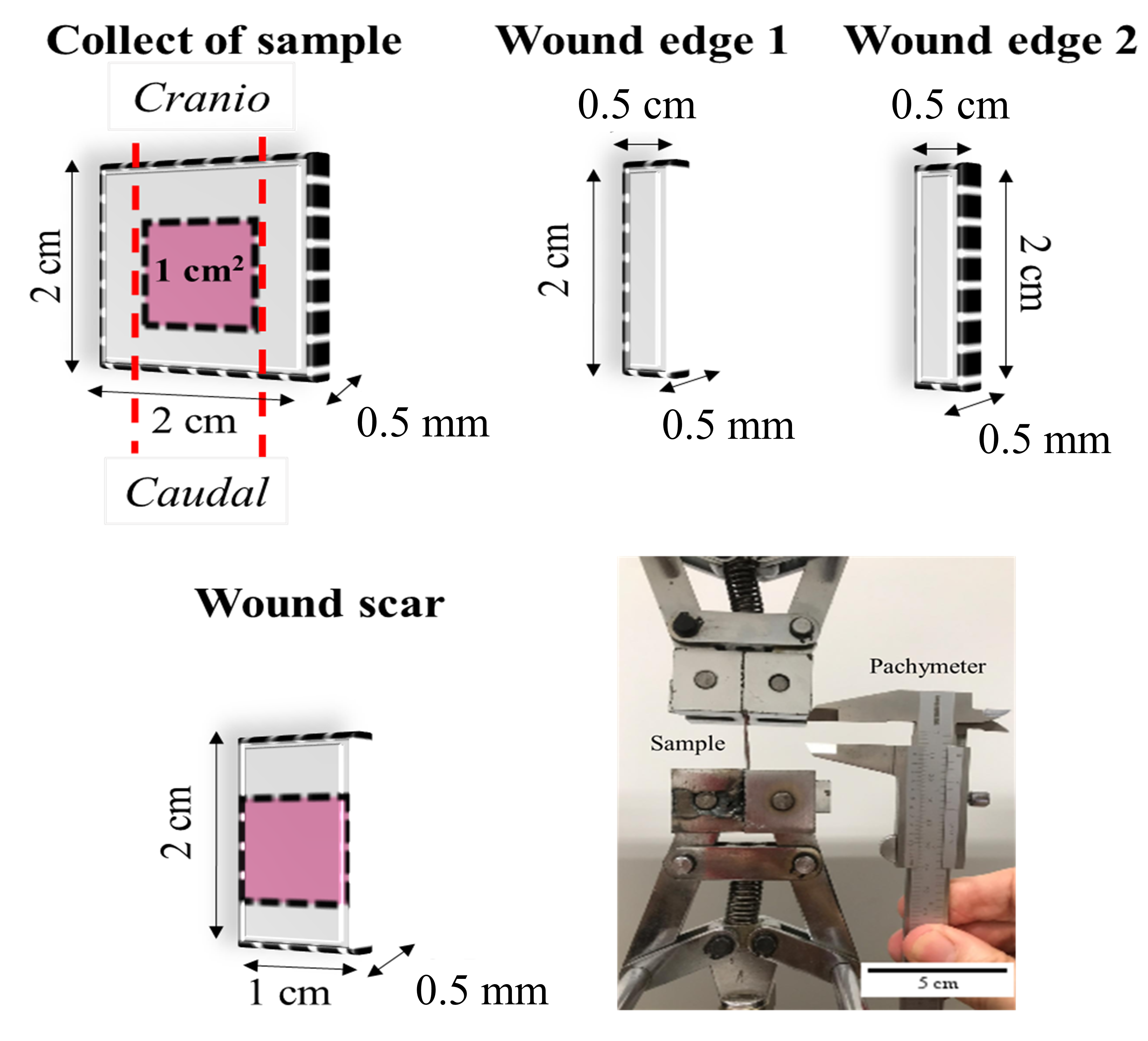
**

**Supplementary figure 2. Dimensions for the traction test on traction machine.** For the traction test, the wound and the edges of each wound were collected (totaling 2 cm^2^) and the samples were sectioned in longitudinal cranio-caudal direction. We analyzed separately two edges of 2 cm x 0.5 cm x 0.5 mm (height, width and thickness) and the wound bed of 2 cm x 1 cm x 0.5 mm. Traction machine with skin sample during the test, beside a pachymeter (5 cm scale)**.**

**Supplementary figure 3. Assessment of fibroblast viability.** Percentage of fibroblasts viability in high glucose medium and fibroblasts cultured in high glucose medium plus 50 µM of EPA or IL-10 (10 ng/mL). Values are expressed as mean ± standard error of the mean (SEM). p <0.05 was considered statistically significant as indicated by Unpaired *t* test. Samples/group: 8 in two independent experiments.


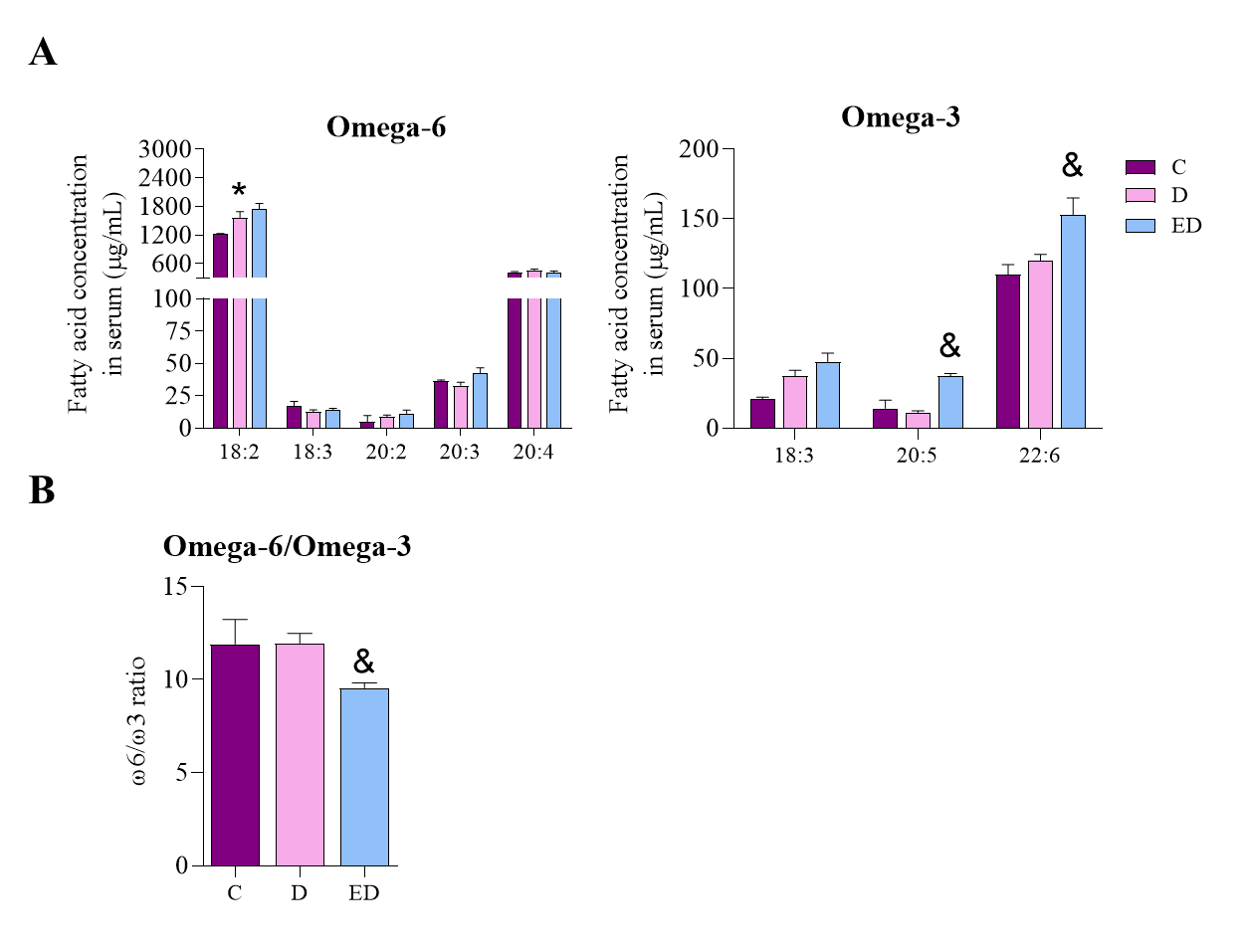


**Supplementary figure 4. Fatty acid composition of serum. (A)** Concentration of fatty acids in serum (µg/mL) harvested from control (C), diabetic (D) and diabetic mice supplemented with EPA-rich oil (ED) 3 days after wound induction. Omega 6: linoleic acid (18:2); γ-linolenic (18:3); eicosadenoic (20:2); dihomo-gamma linolenic (20:3); arachidonic (20:4). Omega-3: α linolenic acid (18:3); eicosatetraenoic acid (20:4); eicosapentaenoic (20:5); docosapentaenoic (22:5) and docosahexaenoic (22:6). Values are expressed as mean ± standard error of the mean (SEM). p <0.05 was considered statistically significant as indicated by Two-Way ANOVA and Bonferroni post-test. (*) C *vs.* D; (&) D *vs.* ED. **(B)** Serum ω-6/ω-3 ratio. Values are expressed as mean ± standard error of the mean (SEM). p <0.05 was considered statistically significant as indicated by One-Way ANOVA and Bonferroni post-test. (&) D *vs.* ED. Animals/group: 7 C, 7 D, 8 ED


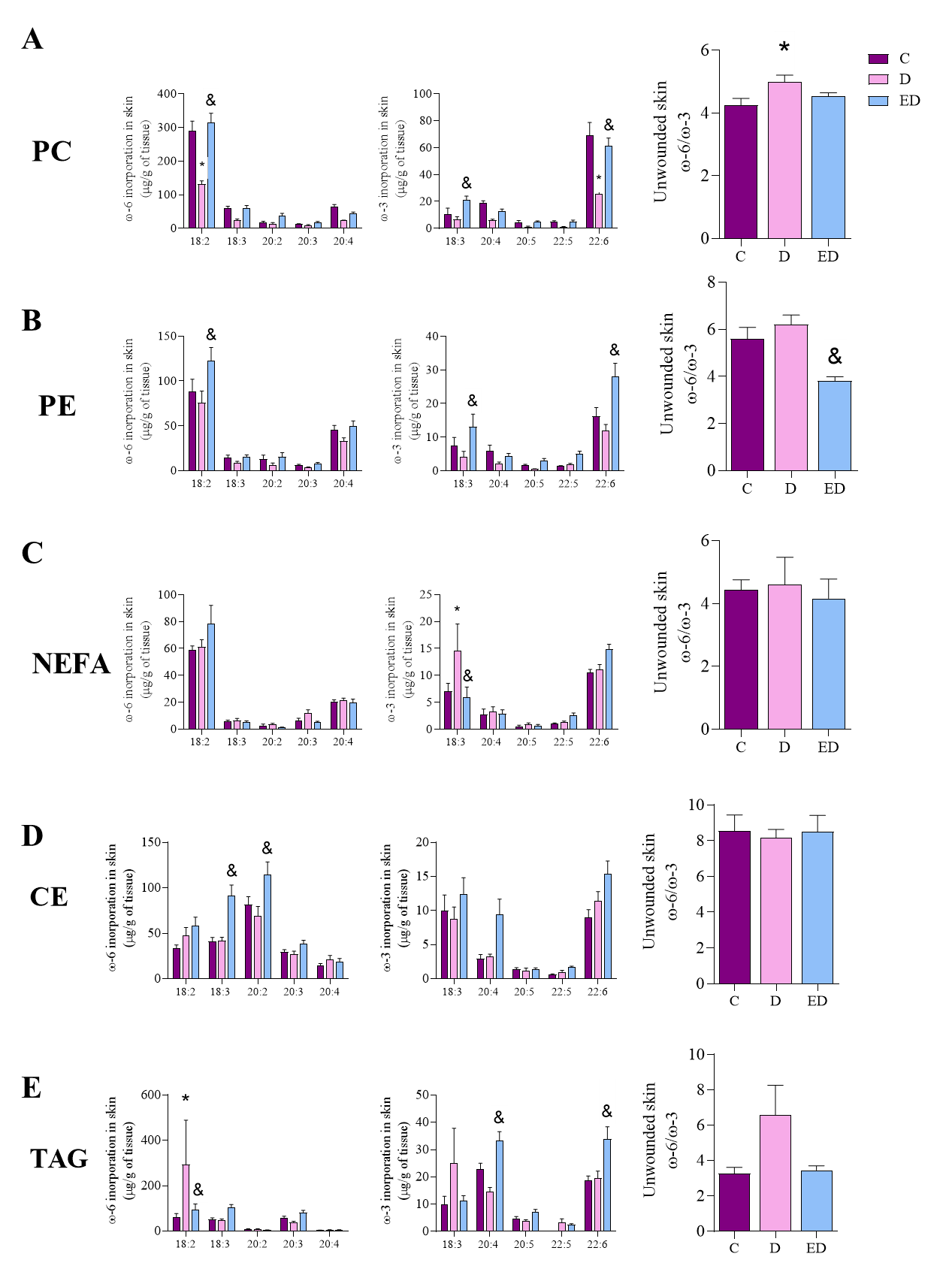


**Supplementary figure 5. Fatty acid composition of skin. (A)** Concentration (µg/g of tissue) of omega-6, omega-3 and omega-6/omega-3 ratio in phosphatidylcholine (PC) fraction in skin harvested from control (C), diabetic (D) and diabetic mice supplemented with EPA-rich oil (ED). **(B)** Concentration of omega-6, omega-3 and omega-6/omega-3 ratio in phosphatidylethanolamine (PE) fraction in skin. **(C)** Concentration (µg/g of tissue) of omega-6, omega-3 and omega-6/omega-3 ratio in non-esterified fatty acid (NEFA) fraction in skin. **(D)** Concentration (µg/g of tissue) of omega-6, omega-3 and omega-6/omega-3 ratio in cholesteryl ester (CE) fraction in skin. **(E)** Concentration (µg/g of tissue) of omega-6, omega-3 and omega-6/omega-3 ratio in triacylglycerol (TAG) fraction in skin. Values are expressed as mean ± standard error of the mean (SEM). p <0.05 was considered statistically significant as indicated by Two or One-Way ANOVA and Bonferroni post-test. (*) C vs. D; (&) D vs. ED. Animals/group: 7 C, 7 D, 8 ED. Omega 6: linoleic acid (18:2); γ-linolenic (18:3); eicosadenoic (20:2); dihomo-gamma linolenic (20:3); arachidonic (20:4). Omega-3: α linolenic acid (18:3); eicosatetraenoic acid (20:4); eicosapentaenoic (20:5); docosapentaenoic (22:5) and docosahexaenoic (22:6).


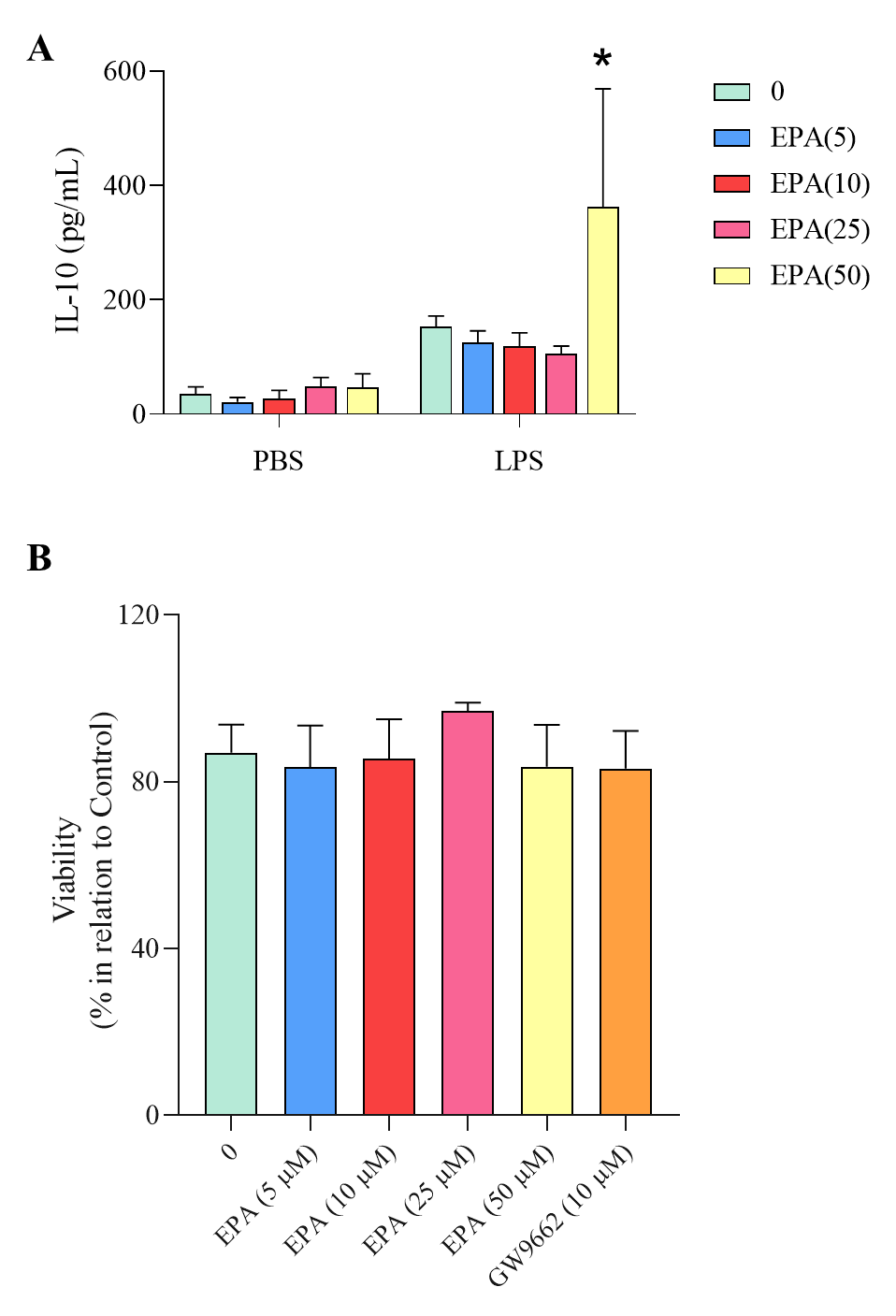


**Supplementary figure 6. In peritoneal neutrophils 50 µM of EPA increase IL-10 production. (A)** IL-10 concentration in supernatant in the absence (PBS) and presence of 1 µg/mL of LPS in different concentrations of EPA (5, 10, 25 and 50 µM). **(B)** Assessment of neutrophils viability. Percentage of viability of neutrophils cultured in high glucose medium and neutrophils cultured in high glucose medium plus 5, 10, 25 and 50 µM of EPA. Values are expressed as mean ± standard error of the mean (SEM). p <0.05 was considered statistically significant as indicated by One-Way ANOVA and Bonferroni post-test. (*) 0 *vs.* EPA (50). Animals/group: 5 in two independent experiments.

**Supplementary table 1.** List of mouse primers used for RT-qPCR

| Target gene | Sequences |
| --- | --- |
| *Col1a1* | CGTCTGGTTTGGAGAGAGCAT |
|  | GGTCAGCTGGATAGCGACATC |
| *Col3a1* | ATGGAGCAAGACAGTCTTTGAATATC |
|  | TCAGGACCCCCAATGTCATAG |
| *Il-10R* | ATTGTGCTGGAAAGCAGGACG |
|  | GAGCAAACGCGACAACATCC |
| *Gpr120* | CTGGGGCTCATCTTTGTCGT |
|  | ACGACGAGCACTAGAGGGAT |
| *Pparγ* | CACAATGCCATCAGGTTTGG |
|  | GCTGGTCGATATCACTGGAGATC |
| *H1R* | CGACACTGTCAGCACCGCCA |
|  | GAAGACAGTCGGAGAGGTCA |
| *H2R* | TGTGGTCGTCTGCCTGGCTG |
|  | CAACACGGGGTACCGCAGTG |
| *B2m* | CCCCACTGAGACTGATACATACG |
|  | CGATCCCAGTAGACGGTCTTG |
| *Ubc* | ACAGACGTACCTTCCTCACCA |
|  | CCCCATCACACCCAAGAACAA |

**Supplementary table 2.** Fatty acid composition of EPA-rich fish oil

| **Fatty acids composition of EPA rich oil** | |
| --- | --- |
| **FAMEs** | **Mean±SD (%)** |
| Palmitic acid (16:00) | 0.13±0.02 |
| Stearic acid (18:00) | 0.70±0.02 |
| Elaidic acid (18:1 trans n-9) | 1.07±0.01 |
| Linoleic acid (18:2 n-6) | 0.22±0.02 |
| γ-Linolenic acid (18:3 n-6) | 0.24±0.02 |
| Linolenic acid (18:3 n-3) | 0.39±0.04 |
| Gondoic acid (20:1 n-9) | 0.69±0.14 |
| Dihomo-gamma-linolenic acid (20:3 n-6) | 0.22±0.06 |
| Arachidonic acid (20:4 n-6) | 5.34±0.20 |
| Eiosapentaenoic acid (20:5 n-3) | 71.35±0.53 |
| Docosahexaenoic acid (22:6 n-3) | 19.65±0.2 |
| **Total saturated** | **0.83±0.03** |
| **Total omega-9** | **1.73±0.15** |
| **Total omega-6** | **6.02±0.30** |
| **Total omega-3** | **91.39±0.78** |

Values ​​presented as mean ± standard deviation of the mean (SD) of three EPA-rich oil samples analyzed by Gas Chromatography Coupled to Mass Spectrometry (GC-MS).

**Supplementary table 3.** Antibodies used in flow cytometry analysis.

| **Antigen** | **Conjugate** | **Source** | **Dilution** |
| --- | --- | --- | --- |
| IL-10 | APC, PE | BD Biosciences | 1:50 |
| CD45 | APCCy7, PECy7, BV570 | BD Biosciences | 1:50 |
| Ly6G | PE, BUV563 | BD Biosciences | 1:50 |
| F4/80 | APCCy7, PercP | BD Biosciences | 2.5:50, 0.5:50 |
| CD117 | APC | BD Biosciences | 1:50 |
| FCeRI | FITC | BD Biosciences | 1:50 |

FITC, fluorescein isothiocyanate; APC,  allophycocyanin; PE, phycoerythrin; APCCy7, APC-Cyanine® 7; PECy7, PE-Cyanine® 7; BV570, brilliant Violet 570; BUV563, brilliant Ultraviolet 563; PercP, peridinin-Chlorophyll-Protein.
